# Biomechanical Evaluation of Spinal Column after Percutaneous Cement Discoplasty: A Finite Element Analysis

**DOI:** 10.1111/os.13314

**Published:** 2022-07-11

**Authors:** Shuang Li, Baoshan Xu, Yancheng Liu, Jingyu Zhang, Guijun Xu, Pengfei Shao, Xiaoye Li, Yongcheng Hu, Xinlong Ma

**Affiliations:** ^1^ College of Orthopaedics Tianjin Medical University Tianjin China; ^2^ Department of Bone and Tissue Oncology Tianjin Hospital, Tianjin University Tianjin China; ^3^ Department of Minimally Invasive Spine Surgery Tianjin Hospital, Tianjin University Tianjin China; ^4^ Department of Neurosurgery Shijingshan Hospital Beijing China; ^5^ The Third Central Hospital of Tianjin Tianjin China

**Keywords:** Biomechanical, Bone cement, Finite element analysis, Lumbar spinal stenosis, Percutaneous cement discoplasty, PMMA

## Abstract

**Objective:**

To compare the biomechanical properties of percutaneous cement discoplasty (PCD) in the spinal column between different implant‐endplate friction.

**Methods:**

A validated L3‐Scarumfinite element (FE) model was modified for simulation. In the PCD model, the L4/5 level was modified based on model 1 (M1) and model 2 (M2). In M1, the interaction between bone cement and endplate was defined as face‐to‐face contact with a friction coefficient of 0.3; in M2, the contact was defined as a Tie constraint. 7.5 N m moments of four physiological motions and axial load of 15, 100 and 400 N preload were imposed at the top of L3. The range of motion (ROM) and interface stress analysis of endplates, annulus fibrosus and bone cement of the operated level were calculated for comparisons among the three models.

**Results:**

The ROM of M1 and M2 increased when compared with the intact model during flexion (FL) (17.5% *vs* 10.0%), extension (EX) (8.8% *vs* −8.8%), left bending (LB) (19.0% *vs* −17.2%) and left axial rotation (LR) (34.6% *vs* −3.8%). The stress of annulus fibrosus in M1 and M2 decreased in FL (−48.4% *vs* −57.5%), EX (−25.7% *vs* −14.7%), LB (−47.5% *vs* −52.4%), LR (−61.4% *vs* −68.7%) and axis loading of 100 N (−41.5% *vs* −15.3%), and 400 N (−27.9% *vs* −27.3%). The stress of upper endplate of M1 and M2 increased in FL (24.6% *vs* 24.7%), LB (82.2% *vs* 89.5%), LR (119% *vs* 62.4%) and axis loading of 100 N (64.6% *vs* 45.5%), and 400 N (58.2% *vs* 24.3%), but was similar in EX (2.9% *vs* 0.3%). The stress of lower endplate of M1 and M2 increased in FL (170.9% *vs* 175.0%), EX (180.8% *vs* 207.7%), LB (302.6% *vs* 274.7%), LR (332.4% *vs* 132.8%) and axis loading of 100 N (350.7% *vs* 168.6%), and 400 N (165.2% *vs* 106.7%).

**Conclusion:**

Percutaneous cement discoplasty procedure could make effect on the mobility or stiffness. The fusion of bone cement and endplate might have more biomechanical advantages, including of the decreasing rate of implant subsidence and dislocation, and the increase spine stability.

## Graphical abstract

Finite element (FE) models of L3‐S used in this article: (A,B) normal model, (C) model 1, the interaction between bone cement and endplate was defined as face‐to‐face contact; (D) model 2, the interaction was defined as a Tie constraint. 15 N m moments of four physiological motions and axial load of 100 and 400 N preload were imposed at the top of L3. The range of motion (ROM) and interface stress analysis of endplates, annulus fibrous and bone cement for the operated level were calculated for comparisons among the three models.

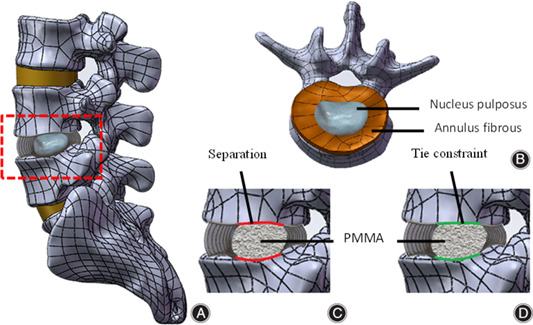

After PCD progress the spine stability was same as normal spine; the implant of bone cement can increase the stress of adjacent vertebrae especially in the upper and lower endplate, and it can be reduced by the way of fusion between bone cement and endplate or fusion.

## Introduction

Lumbar spinal stenosis is prevalent in the elderly people.^1^ Laminectomy and decompression surgery are techniques that have been deemed safe and effective in the treatment of lumbar spinal stenosis.^2^, ^3^ Spinal fusion with fixation using a pedicle screw has been the main surgical strategy for treating lumbar degenerative disc disease (DDD) characterized by unstable spinal segment.^4^ However, traditional open surgery is often limited by severe comorbidities, longer surgery time and damage due to surgical exposure. Minimally invasive procedures are therefore ideal in reducing surgical morbidity and risks of complications.

In 2015, Varga *et al*. reported a new minimally invasive technique named percutaneous cement discoplasty (PCD).^5^ In this technique, polymethyl methacrylate (PMMA) bone cement, which was previously used as a “stand alone” implant, was injected into degenerative interval disc under local anesthesia, to maintain the disc and foramen height, and provide indirect decompression and immediate stability.^6^ Increase instability reduces pain and disability^.^ ^5^

Artificial disc replacement (ADR) or prosthetic disc nucleus device (PDN) are surgical procedures that have been used for the treatment of DDD.^7^ However, the use of the procedures are limited by complications during long‐term follow‐up, including implant dislocation and subsidence, endplate collapse, and low back pain caused by endplate inflammation.^8^, ^9^ These complications are caused by a variety of factors, with the most important being biomechanical factors. In PCD surgery, as a nonabsorbable implant with high elastic modulus, PMMA was injected into intervertebral disc to take the place of nucleus pulposus, which could be regarded as disc or nucleus replacement. However, it is not clear if the complications associated with ADR occur in PCD.

In recent studies^,^ ^4^, ^6^, ^10^ PCD showed ability to alleviate symptoms such as pain in the lower back, lower extremity radiation pain and intermittent claudication in elder patients. However, there were still many issues that need to be addressed: (i) PCD is a novel technique, and mechanical analysis was not carried out during the only short‐term clinical study; (ii) PMMA has a large elastic modulus, which is often higher than cancellous bones of vertebral body in osteoporosis patients. It is therefore unclear if it can cause fracture and end plate collapse during daily activities; and (iii) since PMMA is a type of non‐absorbable implant, it is possible that dislocation of PMMA can happen during long‐term activities. When the friction between PMMA‐endplate interfaces increases, or even fuses, it is not clear if the biomechanical effect of the spine motor unit improves and the collapse and dislocation of implant would be effectively avoided.

To address the above issues, a finite element analysis experiment was designed to simulate the motion unit after PCD surgery. In this study, two contact relationships between bone cement and endplates were simulated: one was sliding friction, reflecting the practical fretting situation; the other was constraint, reflecting an ideal situation with no movement. The present study investigated the biomechanics effects of the PCD procedure on the lumbar vertebrae, including the stress of adjacent segment and the immediate stability of the local column. The aim of study was to enhance the knowledge about the mechanical effects of PCD procedure on lumbar spine stability. First, an effective and accurate finite element PCD model was made to simulate the motion of spine, and the validity of the model was verified. Second, the spine mechanical behavior after PCD procedure was checked and the risk of implanting subsidence and dislocation was accessed. Third, the feasibility of inter body fusion with the method of PCD procedure was evaluated.

## Methods

### Construction and Validation of Normal L3‐Scarum FE Model

**Fig. 1:**
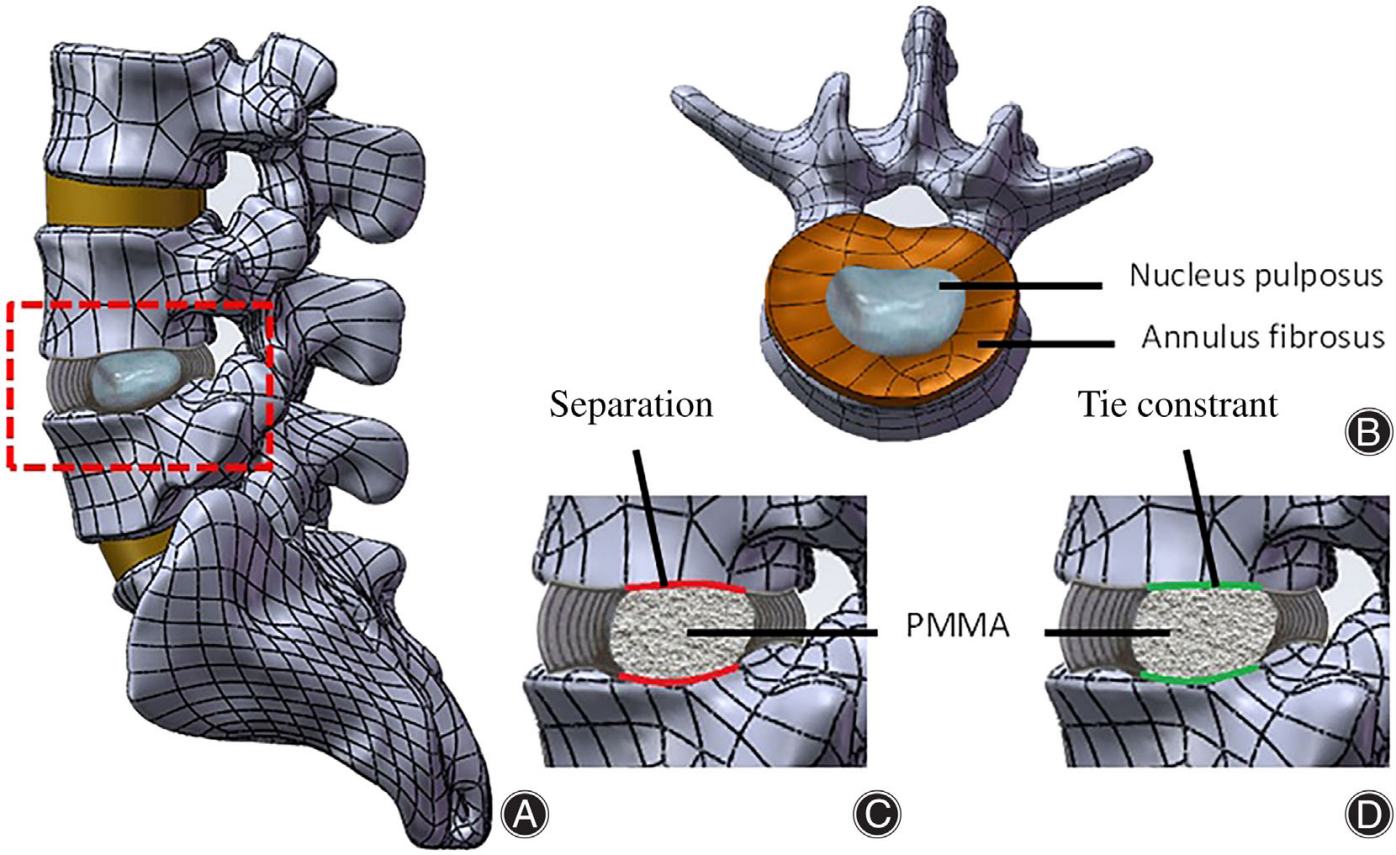
FE models of L3‐S used in this article: (A, B) intact model, (C) model 1, the interaction between bone cement and endplate was defined as face‐to‐face contact; (D) model 2, the interaction was defined as a Tie constraint

A 3‐D FE model of the L3–Scarum lumbar spine was constructed as shown in Fig. 1. Computerized tomography (CT) scan images (0.625‐mm‐thick; Siemens Healthcare, Forchheim, Germany) were acquired from a 27‐year‐old male volunteer. The data obtained was used to create a 3‐D FE model of normal L3‐S lumbar functional segment via the Mimics software (v10.01; Materialise Technologies, Leuven, Belgium). Defeating, smoothening, and amending of the model was done with the Geomagic Studio (v2012; Geomagic Inc., Cary, NC, USA). The Solidworks software (v2017; Dassault Systemes S.A, Boston, MA, USA) was employed to generate the solid model using the cortical bone, intervertebral disc, cancellous bone, as well as the cartilage endplate.

Meshing of the geometric structures was carried out using the ANSYS Workbench (v17; ANSYS Inc., Canonsburg, PA, USA). Each vertebral body was divided into the cortical bone, endplate and the cancellous bone. Simulation of the endplate was done on the superior and inferior surfaces of each vertebra. All bony parts of each lumbar spine component were based on a 10‐node quadratic tetrahedral element (C3D10). The intervertebral disc was portioned into nucleus pulposus and the annulus fibrosus. Nucleus pulposus was shown to constitute 43%^11^ of the overall disc volume and it was located slightly posterior (3.5 mm) to the disc's center.^12^ The nucleus pulposus and the annulus fibrosis were modeled as a homogeneous, hyper‐elastic material using the Mooney–Rivlin model.^13^ All the seven ligaments consisting of ALL (anterior longitudinal ligament), PLL (posterior longitudinal ligament), ITL (intertransverse ligament), LF (ligamentum flavum), ISL (interspinous ligament), SSL (supraspinous ligament), as well as FCL (facet capsular ligament), were created with 2‐node truss elements (T3D2).^14^ The cortical bone and the endplate were 1.0 mm thick^.^ ^14^, ^15^ All the ligament parameters from research evidence and assigned were found to be only tension‐resistant.^16^ The contact of the facet joints was defined as the face‐to‐face contact with a friction coefficient of 0.1.^11^

The mesh was subjected to quality inspection and revised using topological combinations for mesh optimization, and the appropriate mesh densities were determined from a mesh convergence test. Finally, the FE model was made up of 282,293 elements and 493,986 nodes. The element types and element numbers are shown in Table 1.

**TABLE 1:** Mesh information of the FE model

Element set	Element type	Element number
Cortical bone	Tetrahedron (C3D10)	83,348
Cancellous bone	Tetrahedron (C3D10)	178,988
Endplate	Tetrahedron (C3D10)	10,851
Nuclear pulposus/bone cement	Tetrahedron (C3D10)	6333
Annulus fibers	Tetrahedron (C3D10)	2731
Ligaments	Truss (T3D2)	42
Total		282,293

### PCD Models

To simulate the status after PCD process, we removed nucleus pulposus of L4/5 level, and replaced with PMMA bone cement. Based on the different interactions between PMMA and endplates, two contact models (M1 and M2) were simulated:

1. in M1, we defined the interaction between bone cement and endplate as face‐to‐face contact with a friction coefficient of 0.3.^17^ and
2. in M2, this interaction was defined as a Tie constraint.

The contact between the bone cement and annulus fibrosus was termed as separation (Fig. 1). Material properties were described the as previously reported and are shown in Table 2.^18^, ^19^, ^20^

**TABLE 2:** Material properties of components

Element set	Young's modulus (MPa)	Poisson's ratio (μ)
Ortical bone	12,000	0.3
Cancellous bone	100	0.3
Bone cement (PMMA)	2500	0.3
Endplate	1000	0.4
Nucleus pulposus	1.0	0.4999
Annulus fibrous	4.2	0.45
Ligaments		
Anterior longitudinal ligament	15	
Posterior longitudinal ligament	10	
Ligamentum flavum	10	
Supraspinous ligament	8	
Interspinous ligament	10	
Transverse ligament	10	
Capsular ligament	7.5	

### Experimental Test

**Fig. 2:**
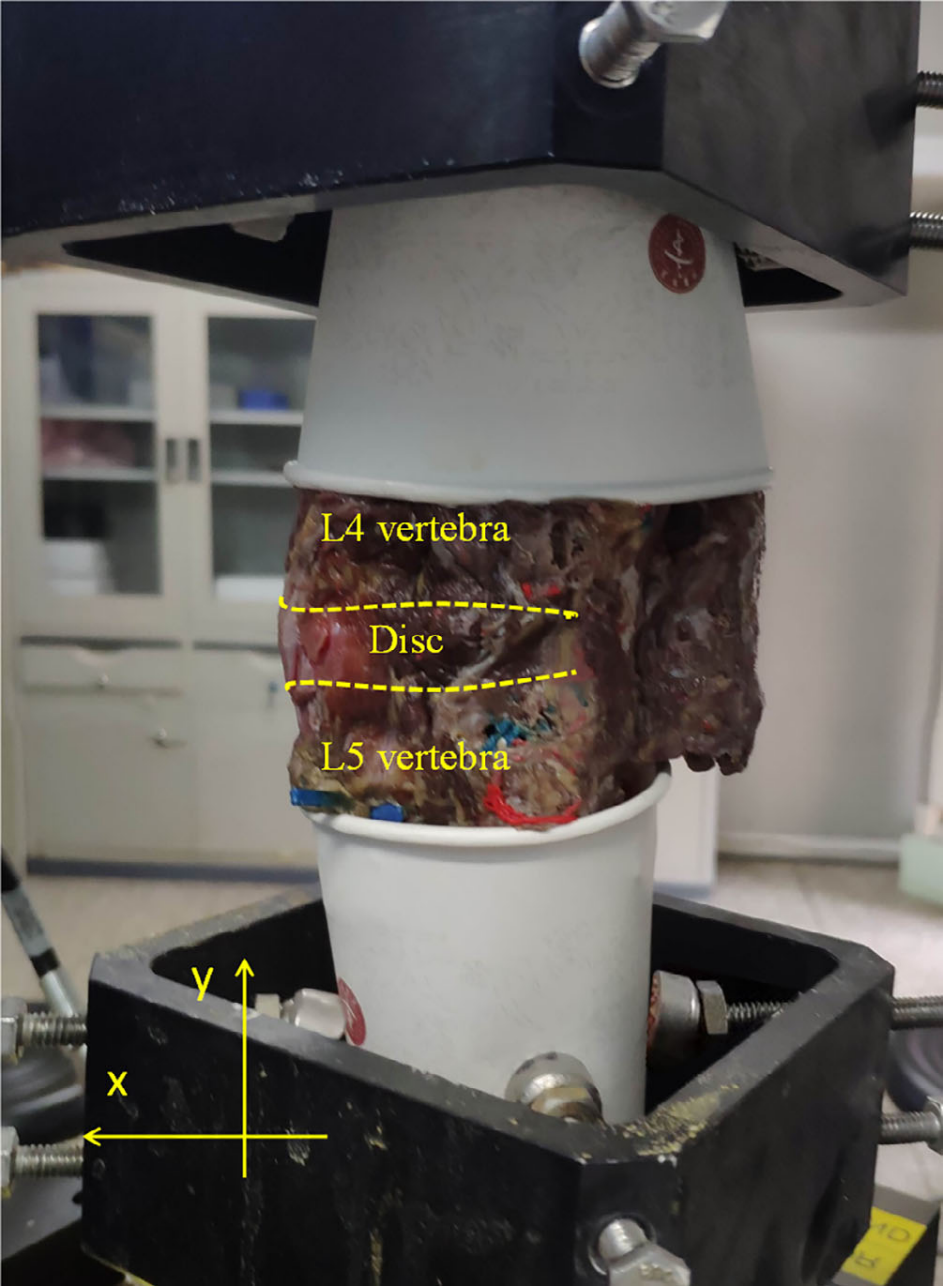
MTS Bionix® Servohydraulic Test Systems and the cadaveric vertebral column specimens of L4/5

To validate the FE model, eight cadaveric vertebral column specimens of L4/5 were stripped of muscle tissue, while preserving the spinal ligaments and facet joints, and were mounted on a MTS Bionix® Servohydraulic Test Systems (Eden Prairie, Minnesota, USA) for biomechanical testing (Fig. 2). The specimens were subjected to flexion, extension, lateral bending and torsion with initial axial preloading stress of 15 N and a torque of 7.5 N m, and the load–displacement curves under different loading conditions were recorded. Three cycles were carried out for each loading state and the 3rd reading was taken as the experimental result to eliminate the time effects such as relaxation and creep of specimens. The mean value and standard deviation were recorded. 15 points (from 0.5 to 7.5 N m with an interval of 0.5) were selected from the load–displacement curves obtained from the biomechanical experiment and the FE model. The root mean squared error (RMSE) was used for error analyses of our model.

### Boundary and Load Condition

A 7.5 N m moment was applied to simulate the model in four directions, including extension (EX), flexion (FL), left bending (LB) and left axial rotation (LR). An axial compressive load of 15 N, 100 N, and 400 N was set on the center of the superior surface of the L1 vertebral body, with the inferior endplate of the sacral vertebra strictly fixed, to simulate resting state, normal activity and aggravating activity, respectively. The range of motion (ROM) of L4/5 level and Von Mise stresses in cement‐endplate interfaces were calculated.

## Results

### Model Validation

**Fig. 3:**
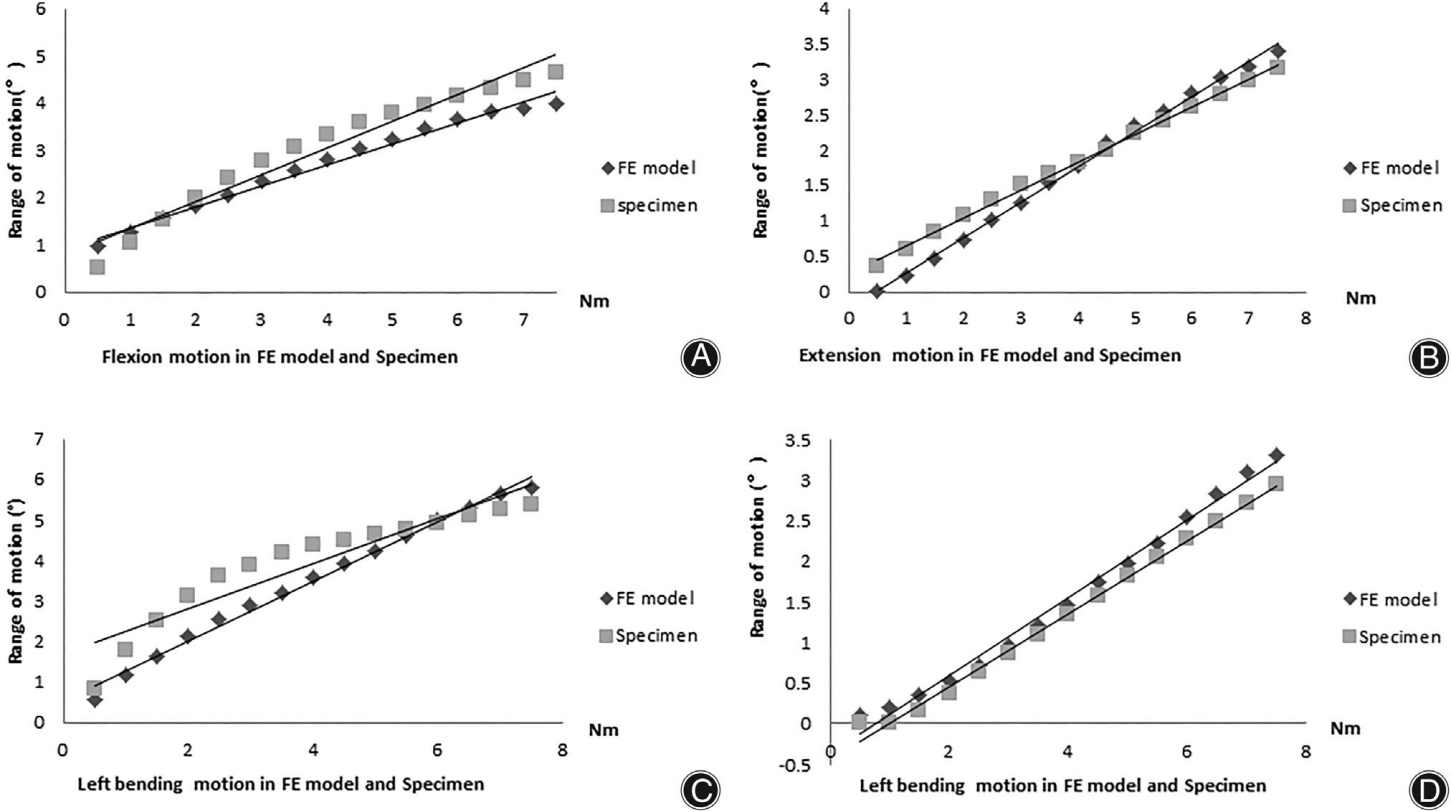
L4/5 level range of motion in flexion (A), extension (B), left bending (C) and left rotation (D) motions between FE model and the experimental data. The trend line shows a similar growth trend between the two groups of data

The ROMs at the L4/5 level in the experimental test are shown in Fig. 3. The RMSE in FL, EX, LB and LR was 0.47, 0.24, 0.68 and 0.22, respectively, indicating good agreement between the FE model and the experimental data, and that the FE model was applicable for predicting real spine biomechanical behavior.

### Range of Motion

**Fig. 4:**
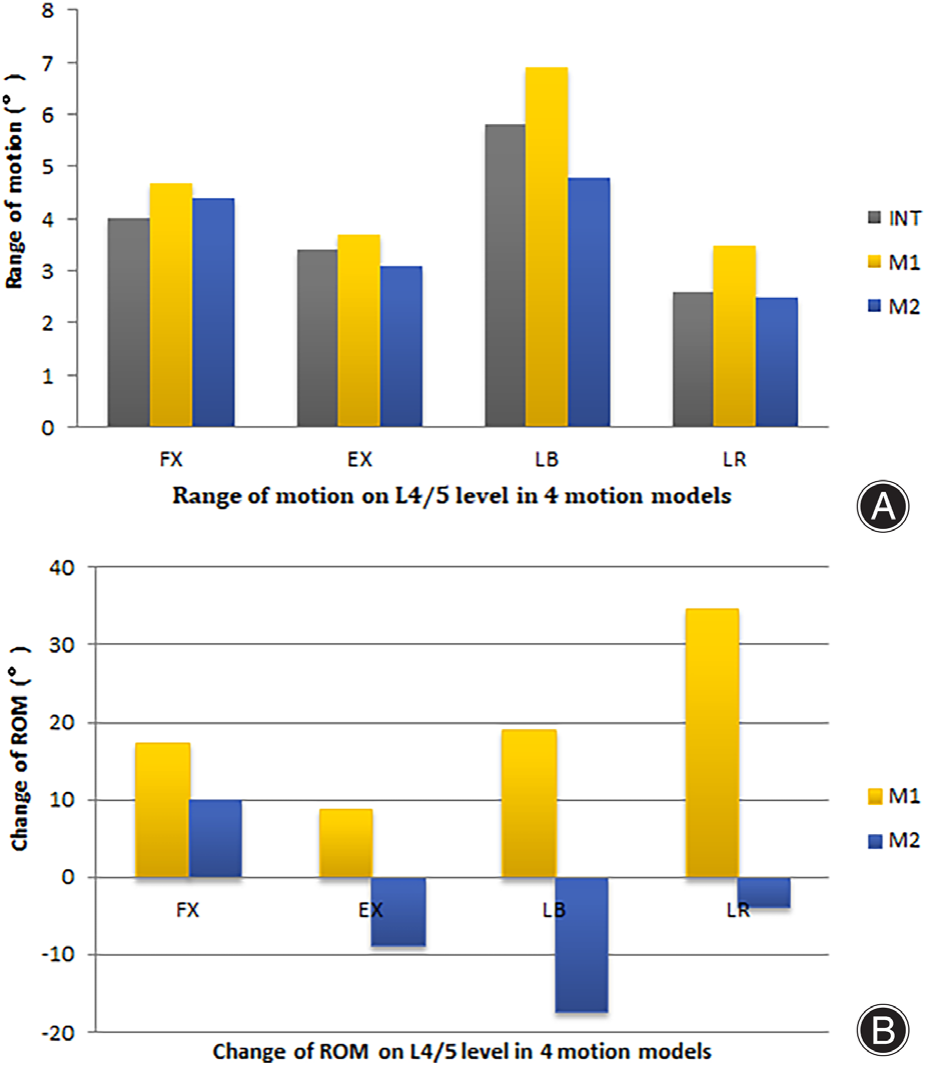
The range of motion (A) and the change of ROM comparing with the intact model (B) on L4/5 level in the motion models of flexion, extension, left bending and left axial rotation

Under the axial compressive load consisting of integration of 15 N and 7.5 N m, ROM was intact, and the treated models are given in Fig. 4.

Compared with the intact model, alterations in the ROMs at the L4/5 level in M1 versus M2 under the four motion states were: FL (17.5% *vs* 10.0%), EX (8.8% *vs* −8.8%), LB (19.0% *vs* −17.2%) and LR (34.6% *vs* −3.8%).

### The Maximum Stress on Annulus Fibrosus and Bone Cement of L4/5 Level

**Fig. 5:**
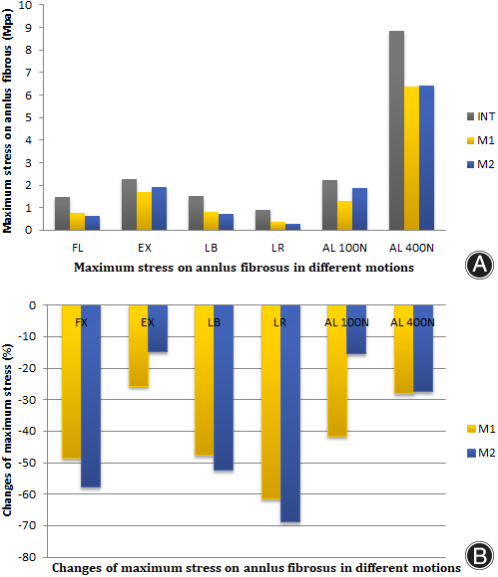
Maximum stress (A) and changes of maximum stress comparing with the intact model (B) on annulus fibrosus in the motion models of flexion, extension, left bending, left axial rotation and axial load of 100 and 400 N

**Fig. 6:**
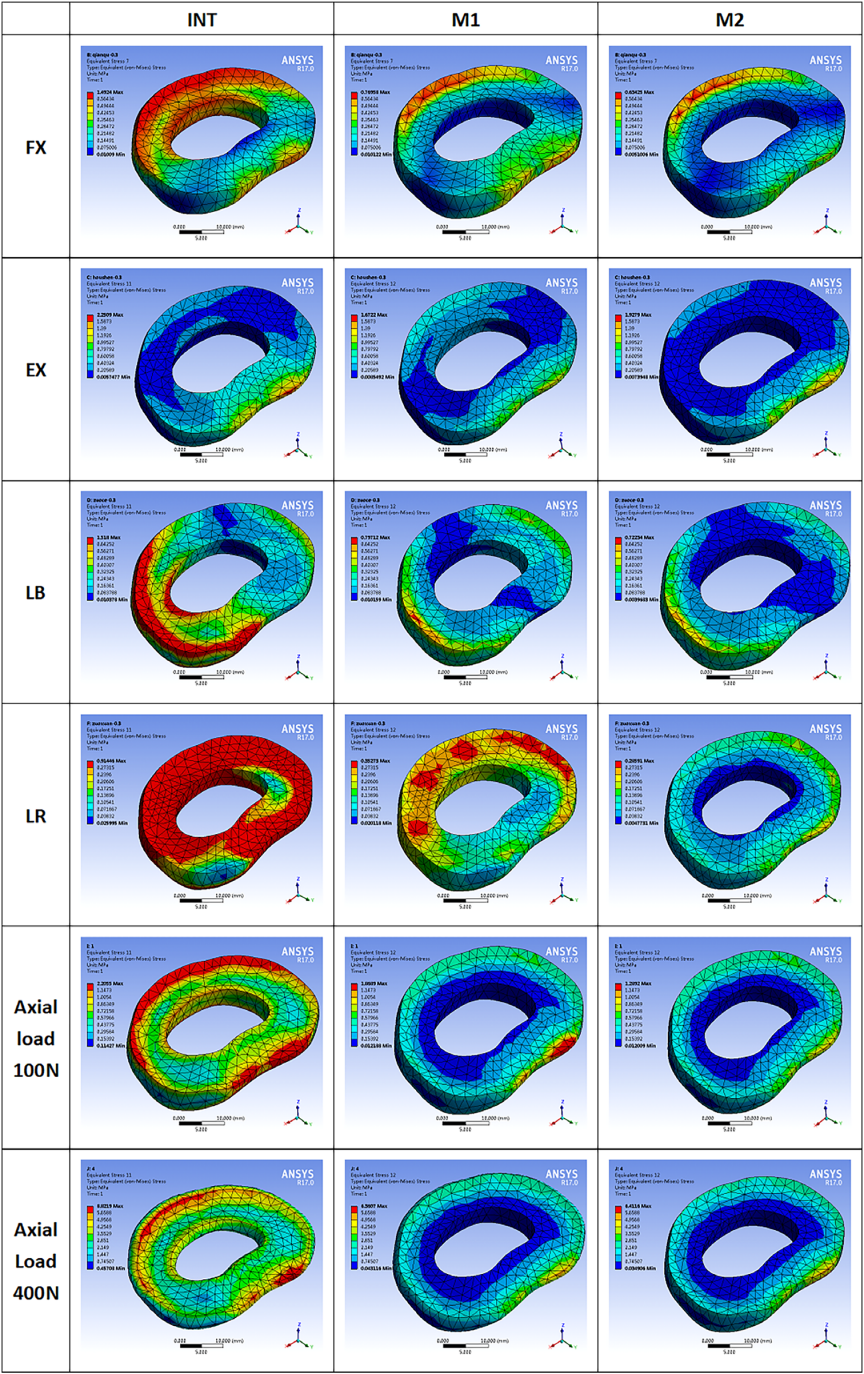
Cloud map of intradiscal pressure at annulus fibrosus in the motion models of flexion, extension, left bending, left axial rotation and axial load of 100 and 400 N

The maximum stress on annulus fibrosus of L4/5 level is given in Figs 5 and 6. The stress of M1 and M2 on annulus fibrosus was calculated under all conditions. The stress changes of annulus fibrosus in M1 and M2 decreased after the PCD procedure under FL (−48.4% *vs* −57.5%), EX (−25.7% *vs* −14.7%), and LB (−47.5% *vs* −52.4%), LR (−61.4% *vs* −68.7%) axial loading of 100 N (−41.5% *vs* −15.3%) and 400 N (−27.9% *vs* −27.3%). In all motions, the annulus fibrosus stress on M1 and M2 decreased when compared with the intact model. Maximum stress of M1 was superior to M2 in FL, LB and LR, but inferior in EX and axial loading of 100 N. The stress in M1 and M2 was almost similar in axial loading of 400 N. Analysis of the cloud map revealed that the maximum stress in the same area on M1 was greater than on M2, indicating that greater friction in the PMMA‐endplate interface produced smaller and more diffuse stress on the annulus fibrosus. Meanwhile, the annulus fibrosus stress on M1 and M2 was smaller than on intact model, indicating that the injection of PMMA had certain protective effects on annulus fibrosus. In the LR motion, maximum stress focused on the posterior annulus fibrosus and increased significantly. In contrast, there was little increase in stress distribution on the posterior annulus fibrosus of M1 and M2.

**Fig. 7:**
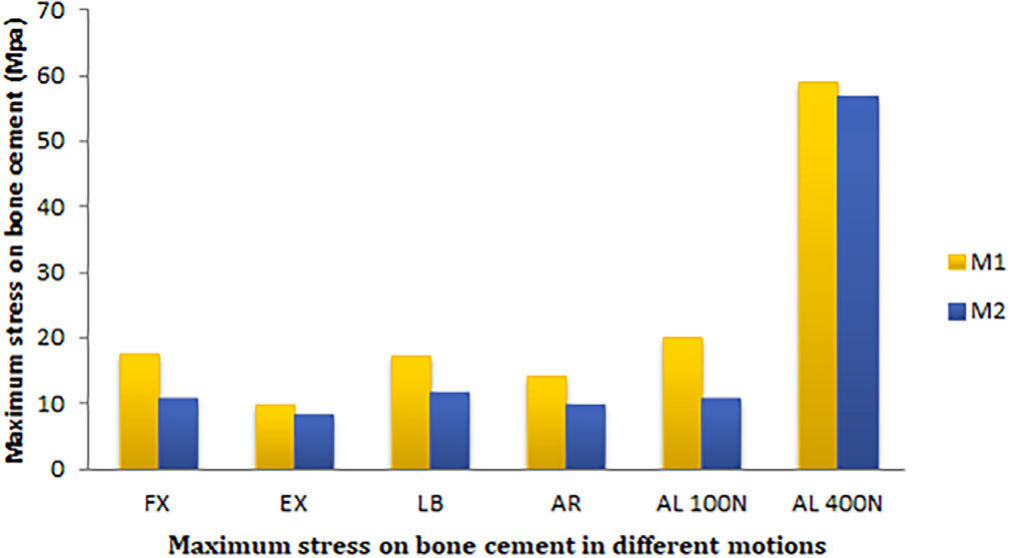
Maximum stress on bone cement in the motion models of flexion, extension, left bending, left axial rotation and axial load of 100 and 400 N

**Fig. 8:**
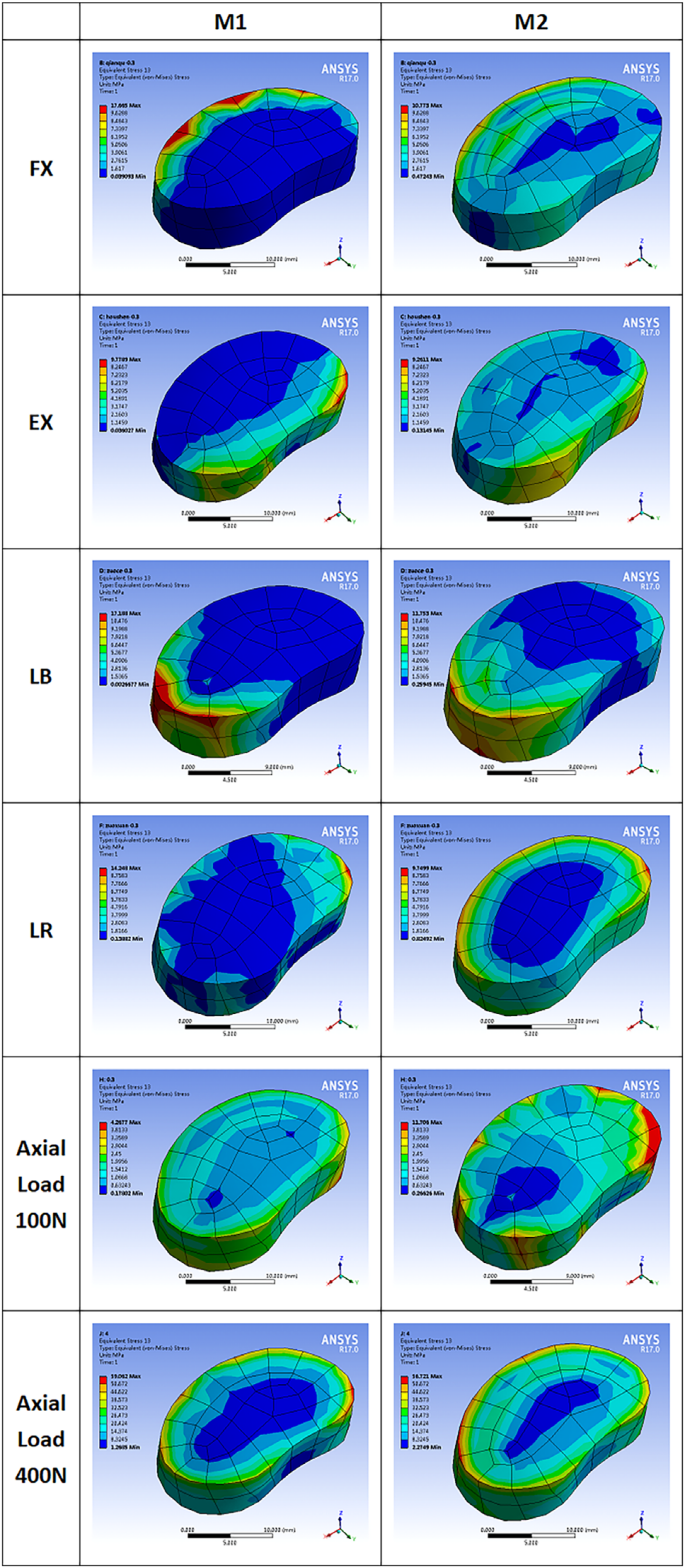
Cloud map of stress in the superior bone cement‐endplate interface for in the motion models of flexion, extension, left bending, left axial rotation and axial load of 100 and 400 N

The maximum stress on bone cement is given in Figs 7 and 8. The stress of M1 and M2 on bone cement was calculated under all conditions. The stress of M1 and M2 decreased after the PCD procedure under FL (17.7 *vs* 10.8 MPa), EX (9.8 *vs* 8.3 MPa), LB (17.2 *vs* 11.8 MPa), LR (14.2 *vs* 9.7 MPa), and axial loading of 100 N (20.2 *vs* 10.9 MPa) and 400 N (59.1 *vs* 56.7 MPa). The stress on bone cement was well correlated for each model. In all conditions, M2 produced less bone cement stress than M1. Compared with M1, the stress on bone cement was well correlated for each model. M2 produced stress that was more evenly distributed and the area of high stress concentration was significantly smaller.

### The Maximum Stress in the Endplate‐Cement Interface at the Treated Level

**Fig. 9:**
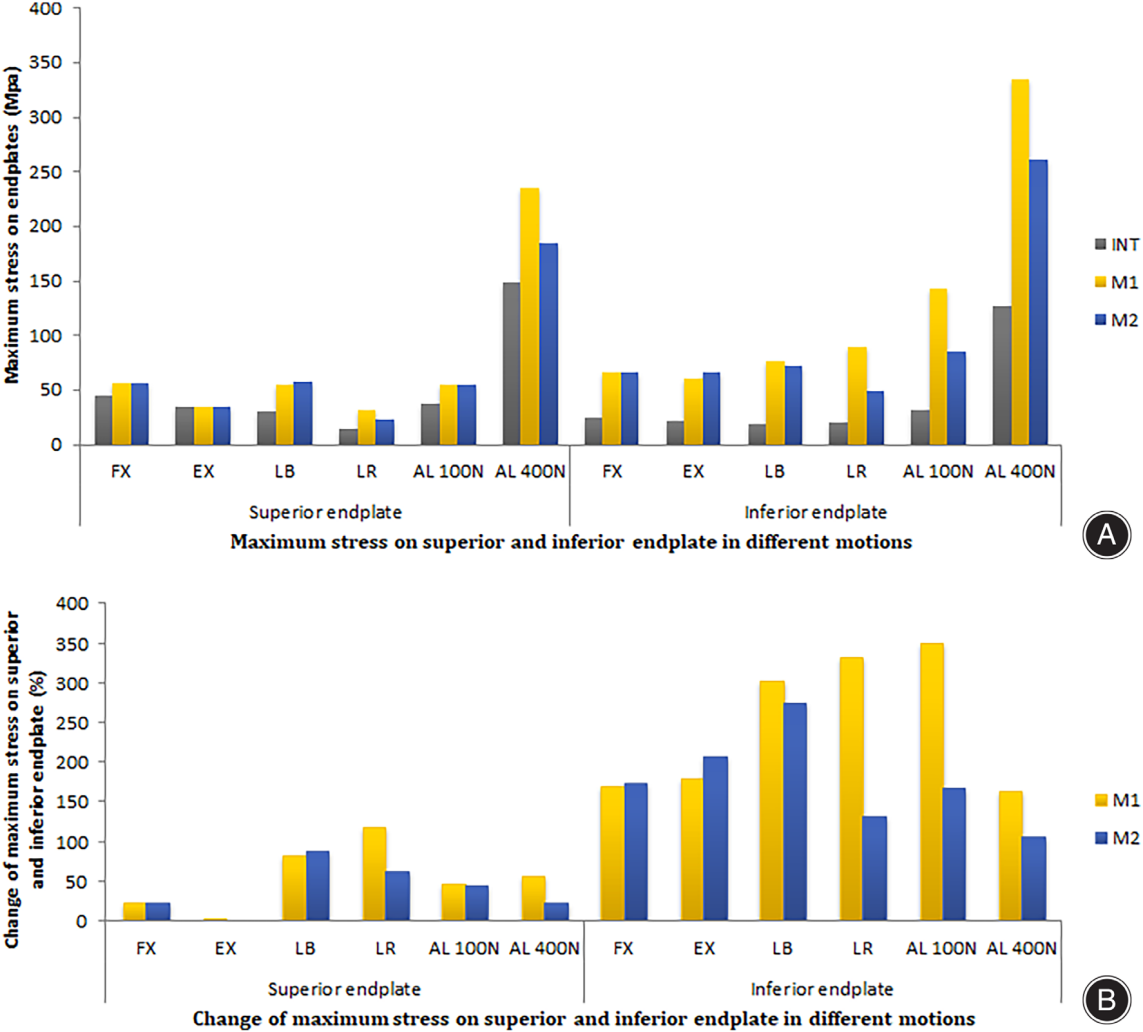
Maximum stress (A) and changes of maximum stress comparing with the intact model (B) on superior and inferior endplate in the motion models of flexion, extension, left bending, left axial rotation and axial load of 100 and 400 N

**Fig. 10:**
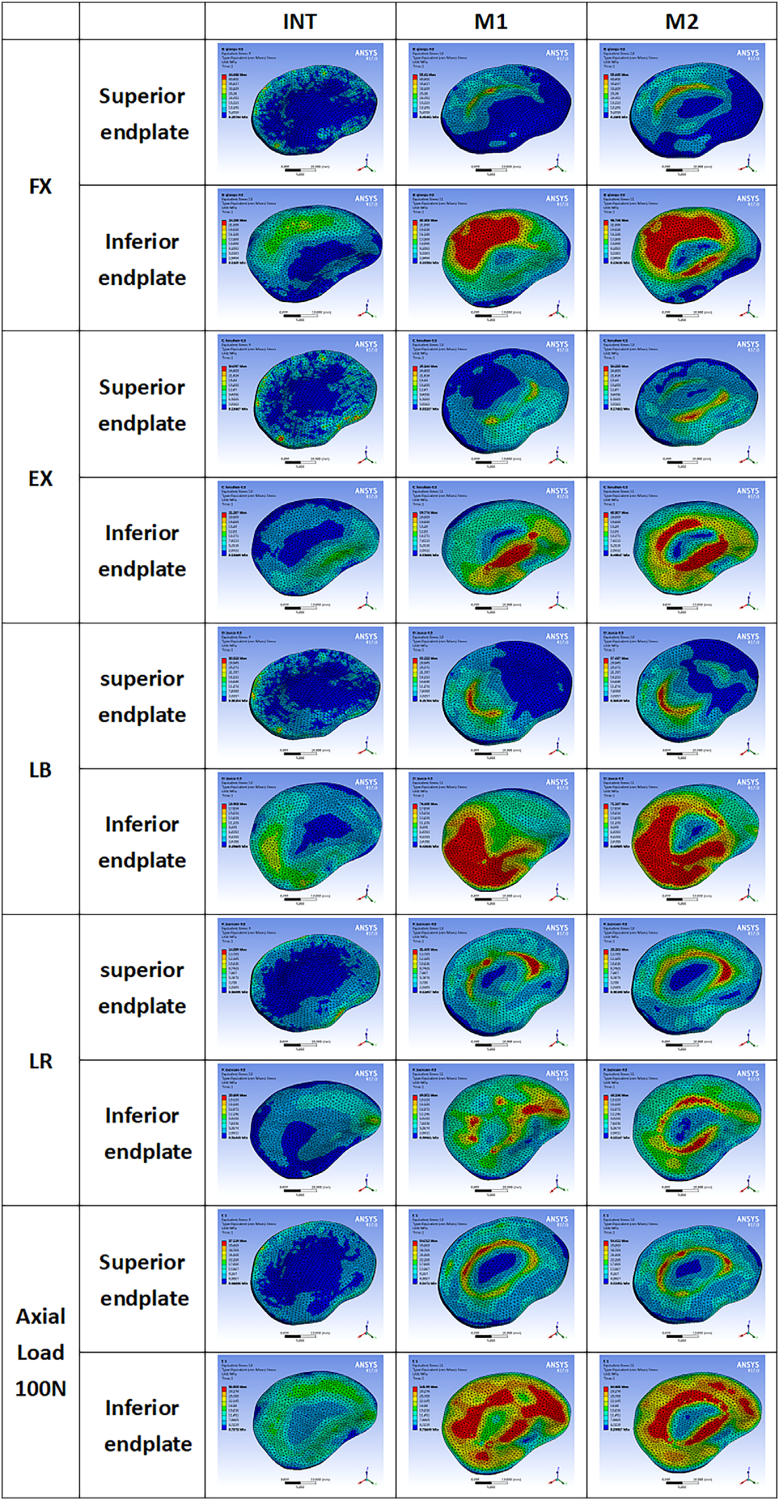
Cloud map of stress in the superior and inferior endplate in the motion models of flexion, extension, left bending, left axial rotation and axial load of 100 and 400 N

The maximum stress on upper endplate is shown in Figs 9 and 10. The stress changes of upper endplate in M1 and M2 increased after the PCD procedure under FL (24.6% *vs* 24.7%), LB (82.2% *vs* 89.4%), LR (119.6% *vs* 62.9%), and axial loading of 100 N (64.6% *vs* 45.5%) and 400 N (58.2% *vs* 24.3%), but were similar in EX (2.9% *vs* 0.3%). The maximum stress of upper endplate was similar in M1 and M2 in FL, EX and axial loading of 100 N, higher in M2 than M1 in LB and axial loading of 400, and lower in M2 than M1 in LR.

The stress changes of lower endplate in M1 and M2 increased after the PCD procedure in all conditions: FL (170.9% *vs* 175.0%), EX (180.8% *vs* 207.7%), LB (302.6% *vs* 274.7%), LR (332.4% *vs* 132.8%), and axial loading of 100 N (350.7% *vs* 168.6%) and 400 N (165.2% *vs* 106.7%). The maximum stress of the upper endplate was similar in M1 and M2 in LF, higher in M2 than M1 in EX and axial loading of 400 N, but lower in M2 than M1 in LB, LR and axial loading of 100 N.

## Discussion

### The Effect on Stability

The current study demonstrated changes in the structure and biomechanical properties of spine after the injection of bone cement into disc. The ROM of the M1 increased after PCD when comparing with the INT model. The ROM of M2 decreased during the motion of EX, LB and LR except in FL, which confirmed that an increase in friction or even a fusion between cement and endplate contributed to a higher stability.

### The Friction of PMMA‐endplate Interface

In this article, two contact models between bone cement and endplate were simulated: M1 simulated a slideable state in the PMMA‐endplate interface; while M2 simulated a stable state with no movement between the two surfaces, which was considered as a hypothetical fusion state. Since the cement can be individually shaped to adapt to the space of annulus fibrosus when injected in the PCD model, we substituted the Pulpous nuclear into bone cement directly as a stand‐alone intervertebral spacer. Since the friction interface characteristic between PMMA and endplate was still unclear, and the endplate surface was not flattened and smooth, the friction coefficient was set at 0.3, based on a previous study. We then analyzed the segmental stability and strain evolution of the two models after PCD to determine whether the friction between cement and endplate would affect the biomechanics of endplate, implant and annulus fibrosus.

The M2 model has similar biomechanical properties as the oblique lateral interbody fusion (OLIF) with a stand‐alone cage. During the PCD procedure, the implant is injected into the disc, which is less invasive than OLIF. The PMMA can be shaped to fit the nucleus removed, which simplifies the surgical process and makes it less invasive. Future studies are required that focus on increasing friction or achieving fusion through surgery or improvement of material properties.

### Stress on Annulus Fibrosus

Due to the supportive effects of bone cement, the maximum stress on the annulus fibrosus in M1 and M2 was similar but reduced compared with the intact model in all motions. The cloud map (Fig. 6) shows that the stress distribution was relatively uniform and the stress concentration area on the annulus fibrosus was mainly concentrated on the outer annulus fibrosus. There was no stress concentration in the weak part of the posterior annulus fibrosus. The largest stress on the annulus fibrosus was observed in LR motion, while the stress in M2 significantly decreased. This indicates that the bone cement is relatively stable after PCD, and does not squeeze the annulus fibrosus or dislocate from a weak point.

### Stress on Endplate

Percutaneous vertebroplasty (PVP) has been utilized for the treatment of osteoporotic vertebral fractures by introducing bone cement into the vertebrae, and achieved good clinical results. The PCD surgical procedures were invented to overcome bone cement leakage into disc during PVP surgery. Varga *et al*.^5^ observed that the adjacent disc with vacuum phenomenon filled with PMMA, and signs of instability had disappeared on postoperative X‐ray. Grant *et al*.^21^ assessed the stiffness of diverse parts on the endplate and revealed a decreasing trend from the outside to the center of the endplate. If the local stress is greater relative to the limit of the related parts, microfractures occur, resulting in osteolysis along with cage subsidence.^22^, ^23^

In this study, the stress cloud map of cement‐endplate interface (Fig. 10) shows that the maximum stress of upper and lower endplates in M1 and M2 were greater than that of the intact model, while the maximum stress in lower endplates increased more significantly than that in upper endplates. This also confirms that fractures tend to occur at the lower endplate. In the stress cloud map of the lower endplate, the maximum stress of M1 and M2 models was similar in FL, EX and LB, but was lower in M2 than M1 under rotation (3.3‐fold), axial load 100 N (3.5‐fold) and axial load 400 N (1‐fold). Therefore, increased friction or interbody fusion can reduce the maximum stress on the lower endplate and protect lower endplate collapse and implant subsidence. It also means a lower risk of microfractures or osteolysis.

### Limitations

Although some parameters can be studied through in vitro biomechanical tests or clinical studies, some of them cannot be measured directly.^24^, ^25^ Finite element (FE) analysis has been widely used for the evaluation of biomechanical behaviors of implants in lumbar disc^.^ ^14^, ^26^

There were limitations to this study. First, simplification of the FE model of the lumbar spine was done to enhance the efficiency of convergence in the FE study. However, the FE model could only be simulated, so does not fully reflect actual settings of the human body. Additionally, lumber disc degeneration can be due to variations in imaging characteristics. In this study, we simulated PCD procedure in a normal spine for the purpose of data testing and consistency, which may be different from the actual setting. There is need to validate our findings in future biomechanical studies.

## Conclusions

The percutaneous cement discoplasty procedure could improve the mobility or stiffness. The fusion of bone cement and endplate might have more biomechanical advantages, including of the decreasing rate of implant subsidence and dislocation, and the increase spine stability.

## Conflict of Interest

The authors declare that they have no conflict of interest.
